# Development of an Electrochemical Sensor Using a Modified Carbon Paste Electrode with Silver Nanoparticles Capped with Saffron for Monitoring Mephedrone

**DOI:** 10.3390/s22041625

**Published:** 2022-02-18

**Authors:** Georgios Christos Papaioannou, Sophia Karastogianni, Stella Girousi

**Affiliations:** Analytical Chemistry Laboratory, Chemistry Department, Aristotle University of Thessaloniki, 54124 Thessaloniki, Greece; gnpapaio@chem.auth.gr (G.C.P.); girousi@chem.auth.gr (S.G.)

**Keywords:** electrochemical sensors, saffron, silver nanoparticles, voltammetry, urine samples, mephedrone

## Abstract

Mephedrone, also known as 4-methylmethcathinone, is growing into a prominent recreational drug for young people. When it came to detecting mephedrone, limited efforts were made using electrochemical sensors. As a result, this application depicts the fabrication of a new, sensitive, selective, and economical electrochemical sensor capable of detecting mephedrone by using silver nanoparticles capped with saffron produced through electropolymerization to modify carbon paste electrodes (CPEs). Silver nanoparticles (AgNPs) were capped with saffron (AgNPs@Sa) using a green method. AgNPs@Sa were studied using electron scanning microscopy (SEM) and UV-vis spectroscopy. The sensor was evaluated under the optimum condition to determine its analytical features. The results showed that this procedure had a wide linear range, low detection limit and sufficient reproducibility. Furthermore, the sensor posed sufficient stability. Moreover, it was applied in the determination of mephedrone in urine samples, showing the potential applicability of this electrochemical sensor in real sample analysis.

## 1. Introduction

**Figure 1:**
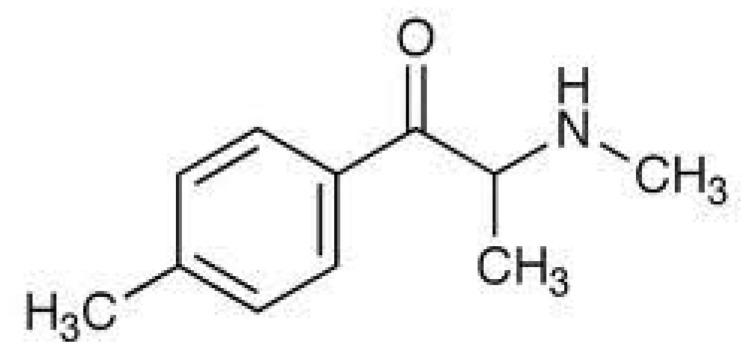
Molecular structure of mephedrone.

Mephedrone, a cathinone derivative Figure 1, is a cheap substitute for conventional drugs and, due to its structures, that can bypass legalization [1]. This drug is predominantly accustomed to combining with other substances to escalate euphoria [2]. Mephedrone is a psychoactive substance with similar effects as 3,4-methylenedioxymethamphetamine (MDMA), methylamphetamine, and cocaine [3]. The molecular structure of the drug is presented in Figure 1. Mephedrone’s effects on humans include feelings of empathy, stimulation, euphoria, appreciation of music, and awareness of senses [4], supporting a sympathomimetic activity of mephedrone [3]. Mephedrone may be snorted, swallowed, or injected [5]. Complications consist of asystole and tremble, high blood pressure, diarrhoea, severe decrease in the diameter of blood vessels, agitation, strokes, nauseousness and regurgitate, cephalalgia, alterations in body temperature, restlessness, inconsequential blackouts, and, in larger dosages, illusions and delirium tremens [6,7]. 

Meanwhile, the electrochemical profile of crocin, the major constituent of saffron (*Crocus sativus* L. (*Iridaceae*)), was studied through cyclic voltammetry using a carbon nanotube electrode [8] or a glassy carbon electrode [9], revealing that crocin has a low oxidation potential and, in this way, is a good electron donor. In addition, random electrochemical studies on carotenoid analogs have shown the formation of a radical cation along the first oxidation step, followed by diamagnetic dications after the second electron transfer [10]. However, saffron is infrequently used as a modifier in electrochemical sensing.

To improve sensitivity and selectivity, electrodes are often modified with carbon-based nanomaterials such as multi-walled carbon nanotubes (MWCNTs) or metal nanoparticles (NPs), for example, AuNPs, PtNPs, and AgNPs [11,12,13]. Due to the novel physicochemical characteristics of AgNPs, alongside their immense electrical and thermal conductivity, they have been used in microelectronics and medical imaging. They offer a larger surface area, higher conductivity, and accelerated electron transfer [12,13,14,15,16].

Recently, analytical methods such as high-pressure liquid chromatography [17,18,19,20,21,22], gas chromatography-mass spectrometry [23,24,25,26], capillary electrophoresis [27], and Raman spectroscopy, as well as surface-enhanced Raman scattering [28] previously dominated the determination of mephedrone. Unluckily, these methods have drawbacks, such as expensive instrumentation, sophisticated procedures, and long analysis time [29]. 

Meanwhile, electrochemical methods and sensors offer the advantages of rapid, portable, low-cost, sensitive, simple, and accurate alternatives for the analysis of forensic drugs and their metabolites, such as mephedrone [12,30]. Moreover, they can be used for their ability to elucidate the action mechanisms of drugs [31,32]. Several studies have already shown the beneficial properties of carbon electrodes regarding the detection of psychoactive substances [33,34,35,36,37,38,39,40,41,42]. These electrodes were also used for mephedrone analysis [43,44]. Mephedrone had already been detected using electrochemical methods [43,44,45,46,47,48,49]. 

Considering the above-mentioned facts, in the present study, we developed an electrochemical sensor using electropolymerized silver nanoparticles capped with saffron as a carbon paste electrode modifier. Briefly, a green method was adopted in the synthesis of silver nanoparticles [50], incorporating sunlight and saffron, which acted as a stabilizer and a reducing agent. The proposed sensor utilized cyclic voltammetry (CV) for the electropolymerization of AgNPs@Sa onto a carbon paste electrode (CPE) surface and adsorptive square wave stripping voltammetry (SWV) measuring the signal of the modified CPE to quantitively determine mephedrone in solution. Finally, the proposed procedure was successfully applied in urine samples, indicating the potential applicability of the sensor to real sample analysis. To the best of our knowledge, this is the first electrochemical sensor that uses silver nanoparticles capped with saffron in the detection of mephedrone. The proposed sensor offers a low detection limit, a broad linear range, short analysis time, low cost, and a sophisticated preconcentration step is not necessary. Thus, the combination of these of saffron and silver nanoparticles ended in the fabrication of a new sensor with high analytical performance capabilities for quantitative measurement of mephedrone in solutions.

## 2. Material and Methods 

### 2.1. Reagents

All reagents were of analytical grade unless stated otherwise, and used as received. The tris hydroxymethyl amino-methane (Tris 99.8%, ACS), ethyl-diamino-tetra-acetic acid (EDTA, ACS reagent, 99.4–100.06%), potassium dihydrogen phosphate, and dipotassium hydrogen phosphate were obtained from Merck (Darmstadt, Germany). Mineral oil (IR spectroscopy), sodium hydroxide, hydrochloric acid, nitric acid, and graphite powder (<20 μm, synthetic) were obtained from Sigma-Aldrich (Saint Louis, MO, USA)). Saffron was purchased from the local market, while silver nitrate was bought by Ducela Biochemie (The Netherlands). Mephedrone was obtained from the National Toxicological Laboratory. Silver nanoparticles were synthesized as previously reported with a slightly different method [50]. All aqueous solutions were prepared with double-distilled water. Stock solutions of 1 g L^−1^ of mephedrone were prepared after weighing a certain amount of the compound and diluting in methanol.

### 2.2. Instrumentation

A Palm Sens potentiostat/galvanostat model 1 was used for voltammetric investigations (Echo Chemie, The Netherlands). The used electrodes in a traditional three-electrode cell were a platinum wire as a counter electrode, a 3 mol L^−1^ KCl saturated Ag/AgCl as a reference electrode, a carbon paste electrode with a 3 mm inner and 9 mm outer diameter of the PTFE sleeve, and a fabricated modified electrode as working electrodes. All the experiments were performed at ambient temperature. The pH of all solutions was measured using a Consort C830 pH meter (Consort bvba, Turnhout, Belgium). 

The electrochemical cells were cleansed with dilute nitric acid and washed with double distilled water. High purity nitrogen was utilized to deaerate the solutions by aspirating the dissolved oxygen for 15 min before each experiment.

### 2.3. Synthesis of Silver Nanoparticles Capped with Saffron (AgNPs@Sa)

Silver nanoparticles were synthesized as previously reported with a slightly different method [50] by adopting a green approach using saffron as a stabilizing agent as well as a reducing agent. Briefly, a certain amount of saffron and silver nitrate of 1:1 mass ratio was dispersed in 25 mL of an aqueous solution of NaOH (pH 10.0) in a 100 mL beaker with stirring, for approximately 5 min, and then exposed to visible light for 30 min in static condition. The colour of the fluid turned from wine red to brownish-yellow, demonstrating that silver nanoparticles had formed. Afterward, the AgNPs@Sa was preserved at ambient temperature, washed with water and ethanol, and dried in a vacuum desiccator before being used to manufacture the intended electrochemical sensor. 

### 2.4. Fabrication of the Electrochemical Sensor and Detection of Mephedrone

The carbon paste electrode (CPE) was fabricated by hand mixing an appropriate amount of graphite and paraffin oil in a mass ratio of 75/25. A segment of the resultant mixture was filled into the PTFE sleeve. The surface was polished smooth by hand on a piece of weighing paper before use. Electrical contact was made through stainless steel screws.

Modified CPE with AgNPs@Sa was fabricated by depositing the nanoparticles on the CPE’S surface (poly-AgNPs@Sa-CPE) using cyclic voltammetry (CV). Thus, cyclic voltametric electrodeposition from −0.300 to + 1.300 V for cycles with a scan rate of 0.30 V s^−1^ and a step potential of 5 mV was performed in the AgNPs@Sa polymerization solution (1 mg L^−1^ AgNPs@Sa in 0.1 mol L^−1^ phosphate buffer pH 7.0 containing 0.03 mol L^−1^ NaNO_3_) to immobilize the polymeric form of silver nanoparticles capped with saffron on the surface of the CPE (poly-AgNPs@Sa-CPE). Then, the electrode was dried at room temperature for 30 min.

The detection of mephedrone was performed by adsorptive transfer square wave stripping voltammetry. Thus, following the electrodeposition of poly-AgNPs@Sa on CPE, the dissolution solution (0.1 mol L^−1^ Tris-HCl buffer pH 9.0) of mephedrone (MCH) was stirred for 180 s without applying a potential for its adsorption to the proposed modified electrode with poly-AgNPs@Sa (MCH-poly-AgNPs@Sa-CPE).

Subsequently, the prepared MCH-poly-AgNPs@Sa-CPE was shipped to the measurement solution (0.1 mol L^−1^ buffer solution pH 4.0 containing 0.05 mol L^−1^ KF), where signal transduction occurred, utilizing adsorptive square wave stripping voltammetry and anodic scanning of the working electrode potential between −0.300 and +1.300 V with a step potential of 15 mV, a pulse potential of 15 mV s^−1^, and a frequency of 4 Hz. The accompanying voltammogram accounted for the oxidation peak of the saffron-capped silver nanoparticles at each measurement, which occurred at approximately +0.300 V.

The raw data were cured with the Savitzky and Golay filter (level 2) of the PalmSens software, followed by a moving average baseline correction with a peak width of 0.03. Replicated measurements were performed after the CPE surface was renewed by cutting and polishing the electrode. All experiments were repeated six times (n = 6), and the average value was used thereafter. 

### 2.5. Determination of Mephedrone in Real Samples

Urine samples came from a volunteer of our laboratory team. Firstly, the urine sample was filtrated. Afterward, it was diluted at 1:100 with double-distilled water and was sonicated for 10 min. Subsequently, filtration with a 45 mm Millipore membrane was performed and the second dilution of 1:25 then took place, and again it remained in the ultrasonic bath for 10 min. Before spiking with standards, a dilution of the urine samples was performed for the background concentration in the blank matrices to be lowered [51]. The solutions were prepared on the day of analysis to avoid oxidation of the mephedrone. The method followed for analyzing the mephedrone’s real sample was the standard addition method. Thus, in each of five volumetric flasks (50 mL) containing urine samples, a known volume of a standard mephedrone’s solution of 1 mg L^−1^ was added. No standard solution was added to the first one. The calibration curve was obtained from the oxidation signal of the above-mentioned solutions. 

## 3. Results and Discussion

### 3.1. Electropolymerization of Silver Nanoparticles Capped with Saffron and Characterization of the Modified CPE

**Figure 2:**
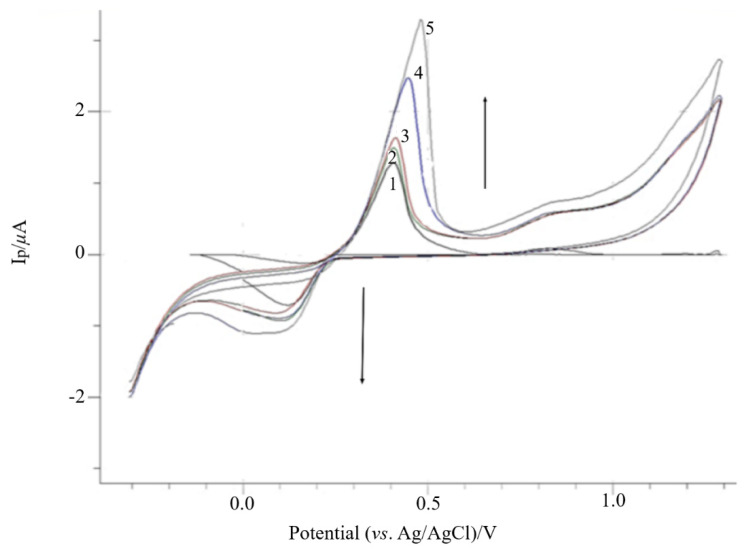
Electrochemical deposition of AgNPs@Sa (428.6 mg L^−1^) in phosphate buffer pH 7.0 containing 0.03 mol L^−1^ NaNO_3_ at 30 mV s^−1^ on CPE. (1) Scan 1, (2) scan 2, (3) scan 3, (4) scan 4, (5) scan 5. Other experimental conditions as mentioned in the Materials and Methods section.

In this study, poly-AgNPs@Sa was potentio-dynamially polymerized on CPE by scanning the potential in the range of −0.300 V to +1.3 V vs. Ag/AgCl and with a scan rate of 30 mV s^−1^. It is worth mentioning that the CPE was not modified and specially cleaned, since it was meant to be used in the preparation of smooth poly-AgNPs@Sa deposits [52,53]. CVs of poly-AgNPs@Sa on CPE, presenting stepwise increases in the peak current over the polymerization process, are displayed in Figure 2. Figure 2 demonstrates that the oxidation peak current increases up to the fifth scan cycle and remains stable above this value, while the peak potential shifts to more positive values. Therefore, it was concluded that an electroactive polymeric film was immobilized on the surface of the CPE.

The formed film gave two irreversible oxidation peaks at approximately +0.451 V and +0.865 V and an irreversible reduction peak at +0.195 V (Figure 2, first potential scan cycle), attributed to the oxidation and reduction of crocin, respectively [8,54]. 

Thus, and according to R. Armellini et al. [54], crocin oxidation takes place through two separate one-electron oxidation processes. Furthermore, the first irreversible oxidation peak at approximately +0.410 V could be assigned to the formation of the free radical species, crocin, Figure 2. Then, the peak current sharply increased, where the crocin radical was further oxidized to crocin^2+^, at +0.860 V, Figure 2. 

Upon reversal of the scan, only a small cathodic wave appeared at +0.190 V, approximately +210 mV less negative than the previous anodic peak, consistent with a pseudo-reversible process [9]. 

To investigate the existence of passivation phenomena in the oxidation process of saffron, saffron (428.6 mg L^−1^) was cycled through 15 scans consecutively. It was found that the difference between the peak current of the first and the last oxidation peak was less than 3%, indicating a negligible passivation effect. Furthermore, by cycling the potential with a scan rate in the range of 4 to 100 mV s^−1^, the resulting peak current varied linearly with the square root of the scan rate, consistent with an oxidation process controlled by diffusion, (ESI, Figure S1) [55].

It was also discovered that the current function *I*p/*γu*^1/2^ (γ mass concentration of saffron) of the oxidation peak declined with the increasing scan rate in the range of mass concentration from 5 to 1000 mg L^−1^ (ESI, Figure S2), indicating the involvement of adsorption in the oxidation reaction [56]. Furthermore, the linear configuration of the *I*p/*γ* vs. *γ* graph at low mass concentrations is in accordance with adsorption in the redox phenomenon (ESI, Figure S3) [56]. 

The peak current intensity was highly reliant on pH, with the maximum being pH = 7.0. Peaks were absent at a pH higher than 8.0. Moreover, the peak potential as a function of pH showed values proximate to those predicted for a proton and two-electron reaction, or that the number of electrons participating in the oxidation was twice that of protons (23.5 mV per pH unit at 25 °C).

On the other hand, the produced film was firm, as it did not drop its response even after one month in the air or after repeated cycles (up to 20 scans) with scan rates between 5 and 50 mV s^−1^. Furthermore, it was not altered by organic solvents such as ethanol, acetone, chloroform, acetonitrile, and dimethyl sulfoxide. These results validate a rigid structure and, in all likelihood, a high molar mass. Instead, the film deteriorated when it was kept in the refrigerator.

**Figure 3:**
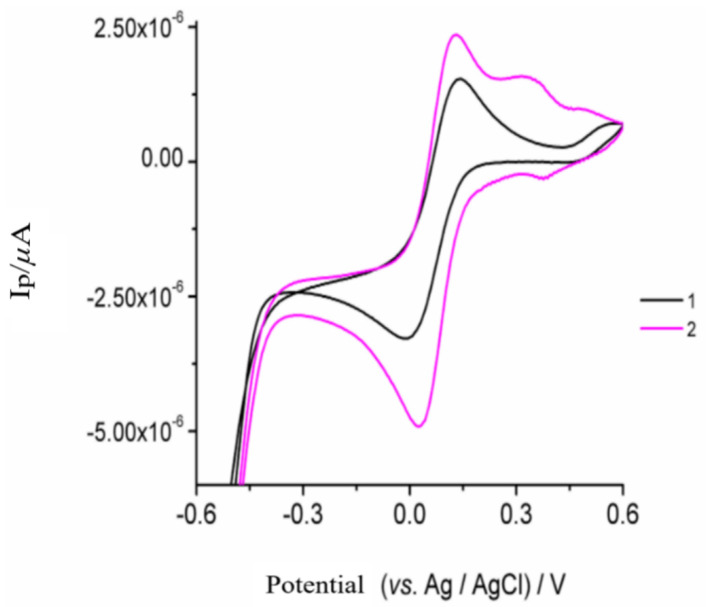
Cyclic voltammograms of 1 × 10^−3^ mol L-1 K3[Fe (CN)6] in 0.2 mol L-1 KCl on the (1) CPE and (2) poly-AgNPs@Sa-CPE, respectively. The conditions are described in the material and methods section.

Cyclic voltammetry was also used to investigate the electrochemical properties of the modified electrode in the presence of 1 × 10^−3^ M K_3_[Fe(CN)_6_] containing 0.200 mol L^−1^ KCl. Here, two defined peaks appeared on the CPE with an anodic peak potential at +0.132 V and a cathodic peak potential at −0.007 V (curve 1, Figure 3). For the poly-AgNPs@Sa-CPE electrode (curve 2, Figure 3), the peak current of the peaks increased, while the value of the peak potential difference between the cathodic and the anodic peak was reduced from +125 mV to +89 mV. This phenomenon may be due to the greater electrochemical activity of the poly-AgNPs@Sa-CPE. The increase in peak current intensity indicated the higher electrochemical activity of the prepared electrode due to its larger electroactive surface [57]. In addition, a pair of low-intensity peaks at an anodic peak potential of +0.362 V and a cathodic peak potential of +0.392 mV were present (Figure 3, curve 2). The electroactive surface area (*A*) of the poly-AgNPs@Sa-CPE electrode (Randles-Sevcik equation) was determined to be equal to 0.022 cm^2^ for CPE, while the electroactive surface area of poly-AgNPs@Sa-CPE was determined to equal 0.067 cm^2^. By comparing these two values, it is evident that the presence of poly-AgNPs@Sa increased the electroactive surface area of CPE. 

**Figure 4:**
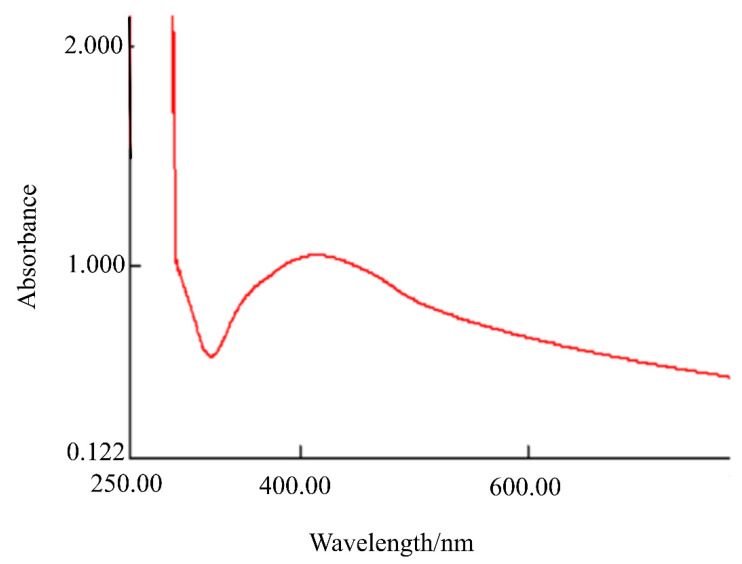
UV-vis spectrum of AgNPs@Sa.

In addition, the formation of AgNPs@Sa was further confirmed by UV-vis spectroscopy. Figure 4 shows the spectrum of AgNPs@Sa. The absorption spectrum of the AgNPs@Sa solution shows two bands at 412.5 nm, Figure 4. The formation of AgNPs@Sa was confirmed by the appearance of this band at 412.5 nm, due to surface plasmon resonance.

### 3.2. Morphology of Sa@AgNPs and Poly-AgNPs@Sa-CPE

**Figure 5:**
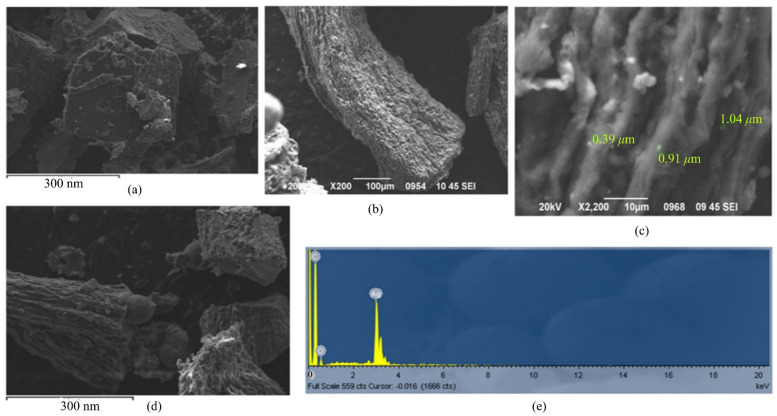
SEM images of (**a**) CPE, (**b**,**c**) AgSa@NPs.in different magnifications, (**d**) poly-AgNPs@Sa, and (**e**) EDX diagram of AgSa@NPs.

The morphological characteristics of CPE (Figure 5a), AgNPs@Sa (Figure 5b,c) poly-AgNPs@Sa-CPE (Figure 5d) were studied through scanning electron microscopy (SEM). As shown in Figure 5b, globular-shaped Ag nanoparticles were synthesized. Figure 5c,d also show the layer particles that had formed organized agglomerates. Furthermore, Figure 5a,d demonstrate morphological differences between the CPE and poly-AgNPs@Sa-CPE, which confirmed the deposition of AgNPs@Sa on the CPE’s surface. The CPE electrode (Figure 5a) is characterized by an irregularly shaped graphite bead with considerable consistency and a small pore size. The polymeric film is homogeneously distributed in the mineral oil on the poly-AgNPs@Sa-CPE electrode with a sufficiently compact structure, similar to the CPE (Figure 5b), but with larger pores. The size of the particles varied, as can be seen in Figure 5c. Smaller sized particles were seen, but their dimension could not be calculated.

The compositional analysis of the AgNPs@Sa was performed by energy-dispersive X-ray (EDX) analysis (Figure 5e). The EDX data (Figure 5e) showed the existence of silver in the synthesized material, confirming the inclusion of the metal within the saffron structure. The silver content reported in the EDX analysis was determined on a small section of the specimen, assuming its uniform composition.

### 3.3. Electrochemical Behavior of Mephedrone on Modified AgNPs@Sa-CPE

**Figure 6:**
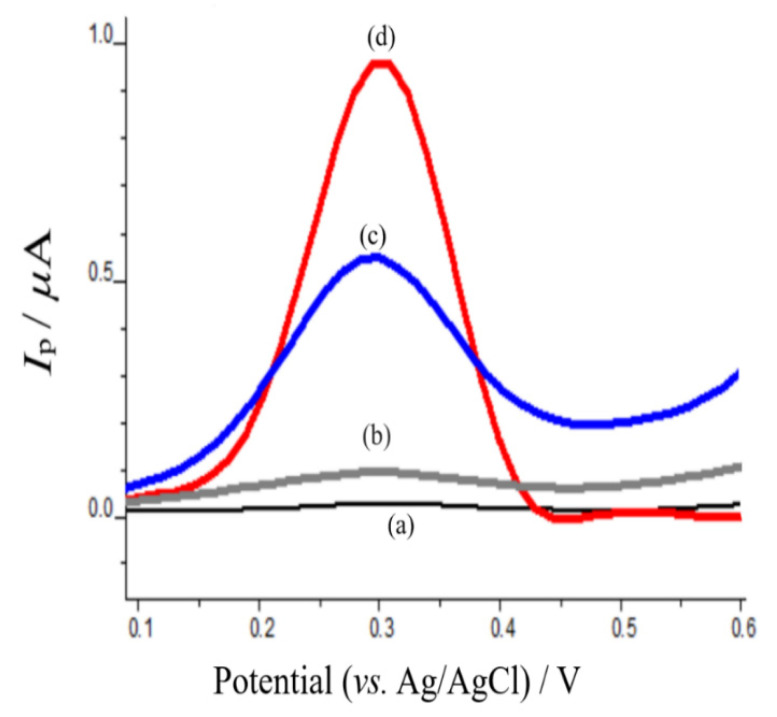
Anodic adsorptive square wave stripping voltammograms of (**a**) CPE, (**b**) 0.04 mg L^−1^ mephedrone on CPE and (**c**) 40.00 mg L^−1^ poly-AgNPs@Sa-CPE, and (**d**) 146.0 mg L^−1^ mephedrone on poly-AgNPs@Sa-CPE in acetate buffer pH 4.0 containing 0.05 mol L^−1^ KF. Other experimental conditions as described in the material and methods section.

The electrochemical properties of mephedrone were investigated with anodic adsorptive stripping voltammetry on CPE and poly-AgNPs@Sa-CPE in pH 4.0 acetate buffer. Figure 6 shows the oxidation square wave voltammograms (SWVs) of poly-CPE (Figure 6a), mephedrone on CPE (Figure 6b), AgNPs@Sa-CPE (Figure 6c), and mephedrone on poly-AgNPs@Sa-CPE (Figure 6d). As can be seen, polyAgSa@NPs (Figure 6c) presented an oxidation peak at approximately +0.300 V in the studied mass concentration. Similarly, mephedrone on CPE also showed an oxidation peak with a low peak current as a response at approximately +0.300 V (Figure 6b). It is worth mentioning, that when mephedrone was studied on the modified CPE with poly-AgNPs@Sa, an oxidation peak was present at +0.300 V, which was larger in its response peak current than those of mephedrone on CPE and poly-AgNPs@Sa (Figure 6d). Therefore, the presence of mephedrone increased the oxidation peak current of the poly-AgNPs@Sa-CPE. These findings are indicative of the possible interaction of mephedrone with AgNPs@Sa and its adsorption on the modified electrode. In summary, the presence of mephedrone enhanced poly-AgNPs@Sa SWV’s response.

According to Armellini et al. [54], the redox behaviour of crocin provides away to express its capacity to donate electrons. Thus, crocin oxidation takes place through two separate one-electron oxidation processes [9,54] and a one proton process. On this basis, electropolymerization of AgNPs@Sa begins with the electrooxidation where the first irreversible oxidation peak at approximately +0.410 V of polyAgNPs@Sa could be assigned to the formation of the free radical species, probably AgNPs-crocin-Sa, where Sa is a saffron molecule with constituents other than crocin [9,54] Then, coupling takes place between AgNPs-crocin-Sa and the AgNPs-crocin^2+^-Sa (formed from the subsequent oxidation of AgNPs-crocin at 0.860 V [54,56]). Aftrewards, termination of the polymerization procedure takes place, and polyAgNPs-[crocin]_n_-Sa is formed. 

Meanwhile, mephedrone did not give any peaks at the studied mass concentrations both on CPE and modified CPE with silver nanoparticles. Schram et al. [58] reports that the oxidation of mephedrone is a two electron and one proton procedure, and presents two redox peaks indicating the oxidation of the secondary amine group and the oxidation of the methylenedioxy group at pH 7.0. The first oxidation peak of mephedrone could be associated to nor-methylone [58]. This product is formed after the oxidation of the secondary amine and subsequent hydrolysis of the imine intermediate [58]. The second peak could be assigned to N,4-dimethylbenzamide [58]. 

It was found that the peak potential of polyAgNPs-[crocin]_n_-Sa in the presence of mephedrone as a function of pH showed values proximate to those predicted for a one proton and two-electron reaction (33.5 mV per pH unit at 25 °C).

Therefore, based on present findings and the literature [9,54,55,56,57,58,59,60], mephedrone was probably, firstly, diffused from the bulk solution to the surface of polyAgNPs-[crocin]_n_-Sa, where subsequent adsorption the mephedrone on the modified electrode took place. Then, the secondary amine moiety of adsorbed mephedrone was oxidized to normethylone, and finally hydrolyzed to N,4-dimethylbenzamide.

## 4. Analytical Performance of the Proposed Assay 

**Figure 7:**
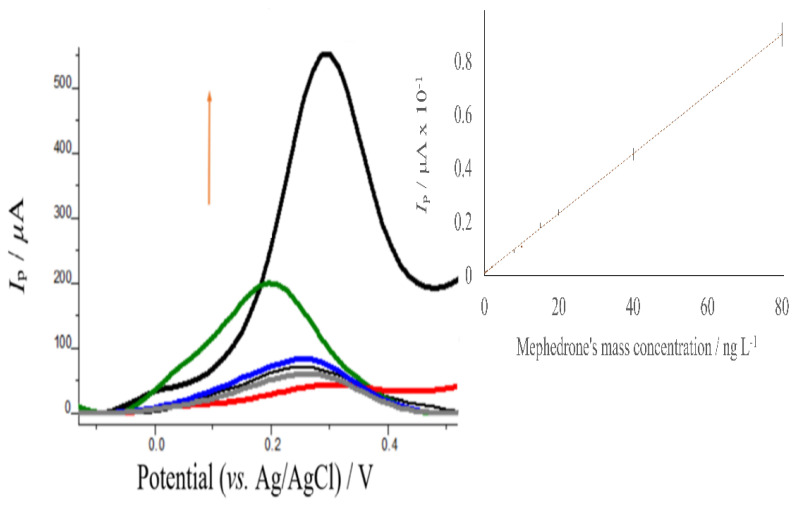
Anodic square wave voltammograms of poly-AgNPs@Sa-CPE under the optimized conditions for mass concentration of mephedrone between 1.841 ng L^−1^ to 80.00 ng L^−1^ (red color: 1.841 ng L^−1^, grey: 3.000 ng L^−1^, black: 5.000 ng L^−1^, blue: 10.00 ng L^−1^, green: 20.00 ng L^−1^, bold black: 80.00 ng L^−1^). Inset: mephedrone’s calibration curve from 1.841 ng L^−1^ to 80.00 ng L^−1^. Conditions: 406.2 mg L^−1^ AgNPs@Sa, polymerization solution: apropriate amount of mephedrone in pH 7.0 phosphate buffer containing 0.030 mol L^−1^ NaNO3, polymerization conditions: 30 mV s^−1^ cyclic voltammetry scan rate, five scans; mephedrone’s immobilization solution: pH 9.0. Tris-HCl buffer, immobilization conditions: time 180 s with stirring; measuring solution: pH 4.0 acetate buffer, measuring conditions: a step potential equal to 15 mV, a pulse potential equal to 15 mV s^−1^, and a frequency equal to 4 Hz.

Under the optimum conditions (see ESI, Tables S1–S4), the developed electrochemical sensor was utilized to determine mephedrone in solution by SWV. The oxidation currents of the sensor increased with the concentration (Figure 7) The calibration graph obtained showed a linear relationship between the SWV peak current and the mephedrone mass concentration from 1.841 ng L^−1^ to 80.00 ng L^−1^, with a correlation coefficient equal to 0.9997. The linear regression relationship from the SWV calibration was *y*(*I*/μA) = (0.0111 ± 0.0001) × (ng L^−1^) (0.0105 ± 0.0004). The detection limit was determined by 3 *s*_b_/slope, where sb is the standard deviation of the blank measurements and the slope of the calibration curve. A detection limit of 0.608 ng L^−1^ was obtained.

The relative standard deviations at 0.500 ng L^−1^ and 5 ng L^−1^ of mephedrone were 4.5% and 4.2%, respectively, indicating favorable reproducibility of the assay (intraday precision). The linear dynamic range for mephedrone in the present study is better than obtained in several previous reports as shown in Table 1. In addition, the detection limit is better than the electroanalytical methods reported in the literature and comparable to other analytical assays, Table 1.

## 5. Determination of Mephedrone in Real Samples

**Figure 8:**
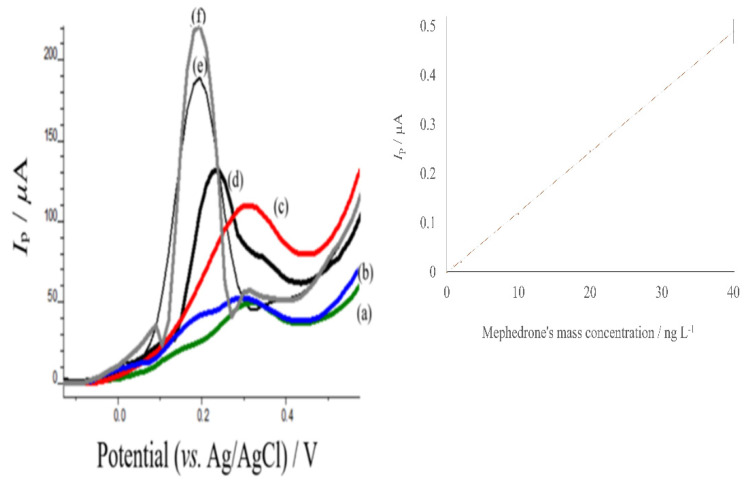
Standard addition method curve of mephedrone in urine samples. (**a**) 0 ng L^−1^, (**b**) 1 ng L^−1^, (**c**) 2 ng L^−1^ (**d**) 10 ng L^−1^, (**e**) 20 ng L^−1^ (**f**) 40 ng L^−1^ of mephedrone in urine samples. Optimum conditions as mentioned in the material and methods section.

The proposed methodology was applied to the determination of mephedrone in urine samples by applying the standard addition method, testing the applicability of the assay in urine samples. Since there was not an available mephedrone consumer, we had to add mephedrone solutions to the prepared urine samples. The regression equation for the urine samples (Figure 8) was found to be equal to I(*μ*A) = (0.0138 ± 0.0001) *γ*_mephedrone_ (ng L^−1^) + (0.00141 ± 0.00135), with a linear correlation coefficient *r*^2^ = 0.9998. The proposed assay exhibited a linear behavior over the concentration range 0.978 to 40.00 ng L^−1^. The LOD (calculated as above) was found to correspond to 0.0757 ng L^−1^. Traditional laboratory assays for mephedrone detection in urine (LOD = 2.000 ng L^−1^) presented considerably lower detection limits than the clinically relevant levels obtained by HPLC-HRqToFMS analysis [59]. It is noteworthy that, within the data, the standard deviation amongst replicates of the same mephedrone concentration in urine samples displayed some differences. While the linear range is indicative of the potential of the proposed sensor to be used in quantitative analysis, there is an appreciable degree of variation in measurements, which would hinder the accurate quantitative determination of mephedrone. Thus, it is better to use this procedure for the confirmation of the presence of mephedrone in mass concentrations greater than 1.841 ng L^−1^ in aqueous samples and 0.978 ng L^−1^ in urine samples. Thus, the developed electrochemical sensor could be a promising tool for mephedrone’s reliable and sensitive tool in clinical analysis. 

The difference slope of the calibration curve in buffer solution and urine spiked samples indicates the influence of the matrix effect. Thus, it was calculated and found to be equal to 0.0027. This value is small and indicative that the matrix effect is insignificant [60].

## 6. Recovery Studies

Standard solutions of mephedrone were added to a sample of urine (to obtain final concentrations of 2, 10, and ng L^−1^). The recovery (the mean of three measurements) and relative standard deviation (*s*_r_) are reported in Table 2. The proposed electrochemical sensor gave 95 to 100% recovery, and *s*_r_ less than 1%. Therefore, the proposed assay showed very good accuracy.

## 7. Conclusions

This paper created and characterized a modified carbon paste electrode with silver nanoparticles capped with saffron. The modified electrode produced square wave signals depending on mephedrone mass concentration in the range 1.841–80.000 ng L^−1^ for the buffer solution and 0.978 to 40.00 ng L^−1^ in the case of a spiked diluted urine sample. The methodology proved to be simple, quick, and economical,. with a low detection limit of 0.978 to 40.00 ng L^−1^ for the buffer and urine samples, respectively. It exhibited very good selectivity, as the compounds with which mephedrone is likely to coexist do not interfere Figure S3. Variation of the oxidation peak current Ip of the polyAgNPs@Sa with the γ mass concentration of polyAgNPs@Sa approaches for detecting and quantifying mephedrone rely on the use of mass detectors, which are costly and cannot be customized for on-site screening. Electrochemical methods, on the other hand, profit from the portable and disposable nature of carbon paste electrodes. 

## Figures and Tables

**Table 1:** Analytical features of selected mephedrone detections.

Detection Method	Electrode	Analytes	Limit of Detection (LOD)	Linear Range	Sample	Reference
LC-MS-MS	-	Mephedrone	3 ng mL^−1^	10–500 ng mL^−1^	Urine, plasma and oral fluid	[21]
GC-MS	-	Mephedrone	2 ng mL^−1^	5–2000 ng mL^−1^	Plasma sample	[24]
GC-MS	-	Mephedrone	5 ng mL^−1^	10–2000 ng mL^−1^	Human blood sample	[25]
Cyclic voltammetry and differential pulse voltammetry	Screen printed graphite electrode	Two metabolites of mephedrone: nor-mephedrone (4-methylcathinone, 4-MC) and dihydromephedrone (4-methylephedrine, 4-MMC-R)	3.97 μg mL^−1^ for 4-MC and 3.64 μg mL^−1^ for 4-MMC-R (PBS, pH 7.0 and 3.0, respectively, differential pulse voltammetry)	40–300 μg mL^−1^ for 4-MC (PBS, pH 7.0) and 15–300 μg mL^−1^ (PBS, pH 3.0) (cyclic voltammetry)	Urine samples	[43]
Square wave voltammetry	sol-gel molecular imprinted polymer, polytyramine, and functionalized multi-walled carbon nanotube@ gold nanoparticles (f-MWCNT@AuNPs)		0.8 nM (142 pg L^−1^)	1 to 10 n mol L^−1^ and 10 to 100 n mol L^−1^	Urine and plasma	[45]
-	Screen-printed graphite electrode		39.8 μg mL^−1^ (pH 2)	16–350 μg mL-1 (pH 2)	Real street samples	[47]
-	Pence British coin		0.56 μg mL^−1^	0.01-0.1 μg mL^−1^	Street samples	[48]
Colorimetric	Portable paper-based Lab-on-a-Chip (LOC) device	Mephedrone	2.51 ng mL^−1^	0.078 to 10.0 mg mL^−1^	Urine	[49]
Adsorptive stripping square wave voltammetry	Silver nanoparticles capped with saffron modified carbon paste electrode	Mephedrone	0.608 pg mL^−1^	1.841–80.000 pg mL^−1^	Urine samples	This work

**Table 2:** Recovery studies of mephedrone in urine samples.

Mephedrone Added/ng L^−1^	Expected Value/ng L^−1^	Measured Value/ng L^−1^	Recovery	*s*_r_
2.00	2.00	1.89 ng mL^−1^	95	1.0
10.0	10.0	9.81 ng mL^−1^	98	1.0
20.0	20.0	19.9 ng mL^−1^	100	0.6

## Supplementary Materials

The following supporting information can be downloaded at: https://www.mdpi.com/article/10.3390/s22041625/s1, Figure S1: Effect of square root of scan rate on the oxidation peak current of poly-AgNPs@Sa-CPE; Figure S2: Variation of the current function *I*p/*γu*^1/2^ of the polyAgNPs@Sa oxidation peak with the increasing of CV’s scan rate; Figure S3: Variation of the oxidation peak current *I*p of the polyAgNPs@Sa with the γ mass concentration of polyAgNPs@Sa; Table S1: Effect of the thickness of the formed film of poly-AgNPs@Sa; Table S2: Effect of the mass concentration of AgNPs@Sa in their polymerization process on CPE; Table S3: Adsorptive transfer square wave stripping voltammetry frequency effect on the detection of mephedrone with the proposed electrochemical sensor; Table S4: Adsorptive transfer square wave stripping voltammetry pulse potential effect on the detection of mephedrone with the proposed electrochemical sensor.
