# Supplementary material for: Development of an Electrochemical Sensor Using a Modified Carbon Paste Electrode with Silver Nanoparticles Capped with Saffron for Monitoring Mephedrone
Source: Sensors (Basel). 2022 Feb 18;22(4):1625. doi: 10.3390/s22041625 (PMC8878875; doi:10.3390/s22041625)
Supplement: Supplementary file 1 [file sensors-22-01625-s001.zip › sensors-1572317-supplementary.pdf]

Electronic supplementary material (ESI)

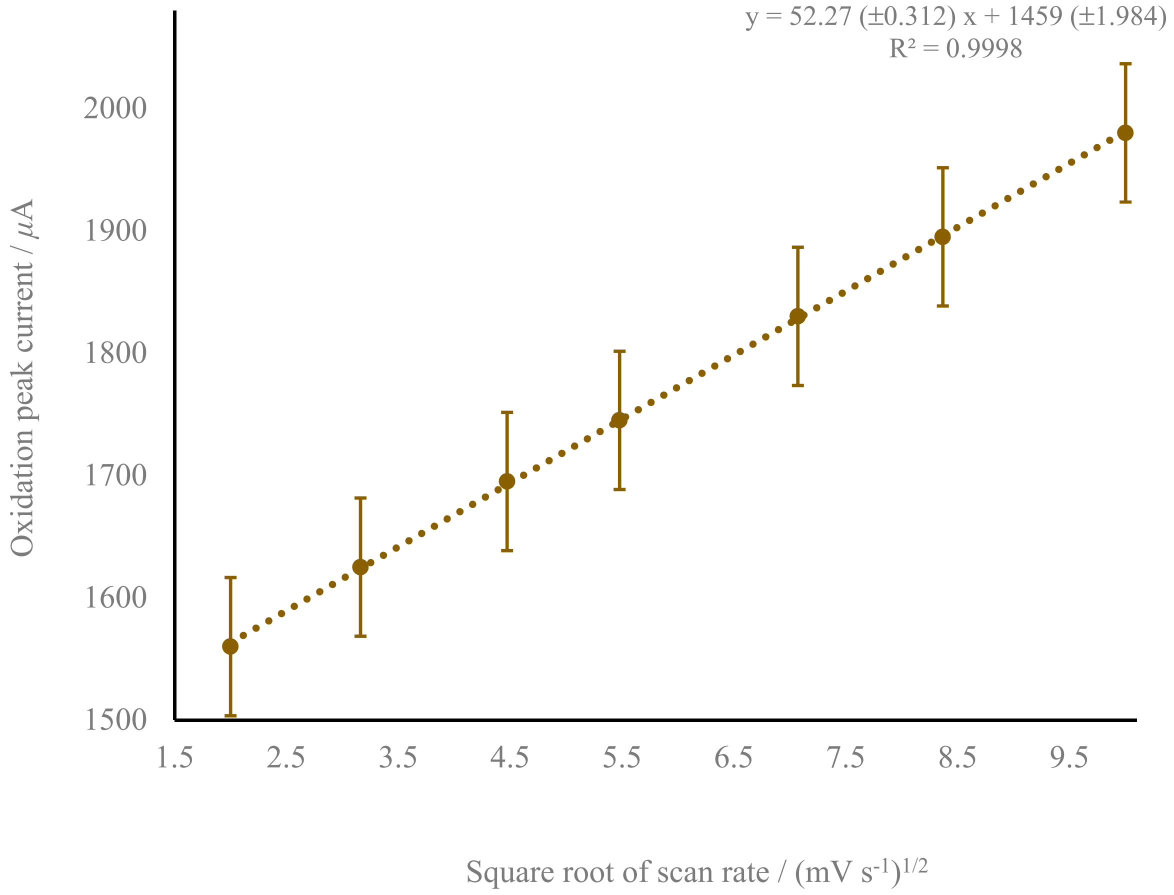

Figure S1. Effect of square root of scan rate on the oxidation peak current of poly-AgNPs@Sa-CPE. Other conditions are mentioned in the experimental section.

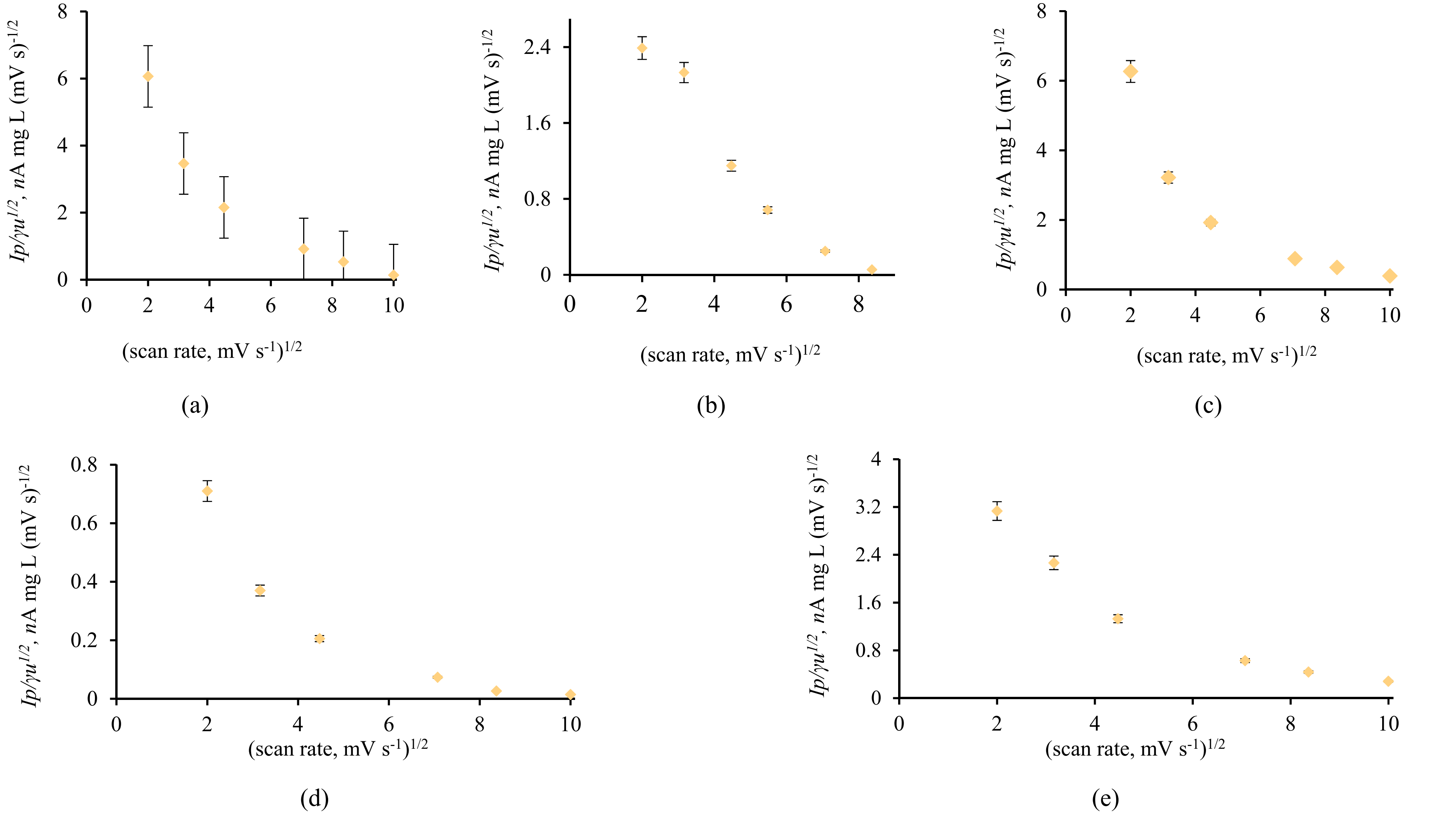

Figure S2. Variation of the current function *I*p/*γu*$^{1/2}$ of the polyAgNPs@Sa oxidation peak with the increasing of CV's scan rate in the range of 4 to 100 mV s^{-1} (a) 5, (b) 50, (c) 100, (d) 500 and (e) 1000 mg L^{-1} of poly poly AgNps@Sa, where is *γ* mass concentration of polyAgNPs@Sa. Other conditions are mentioned in the experimental section.

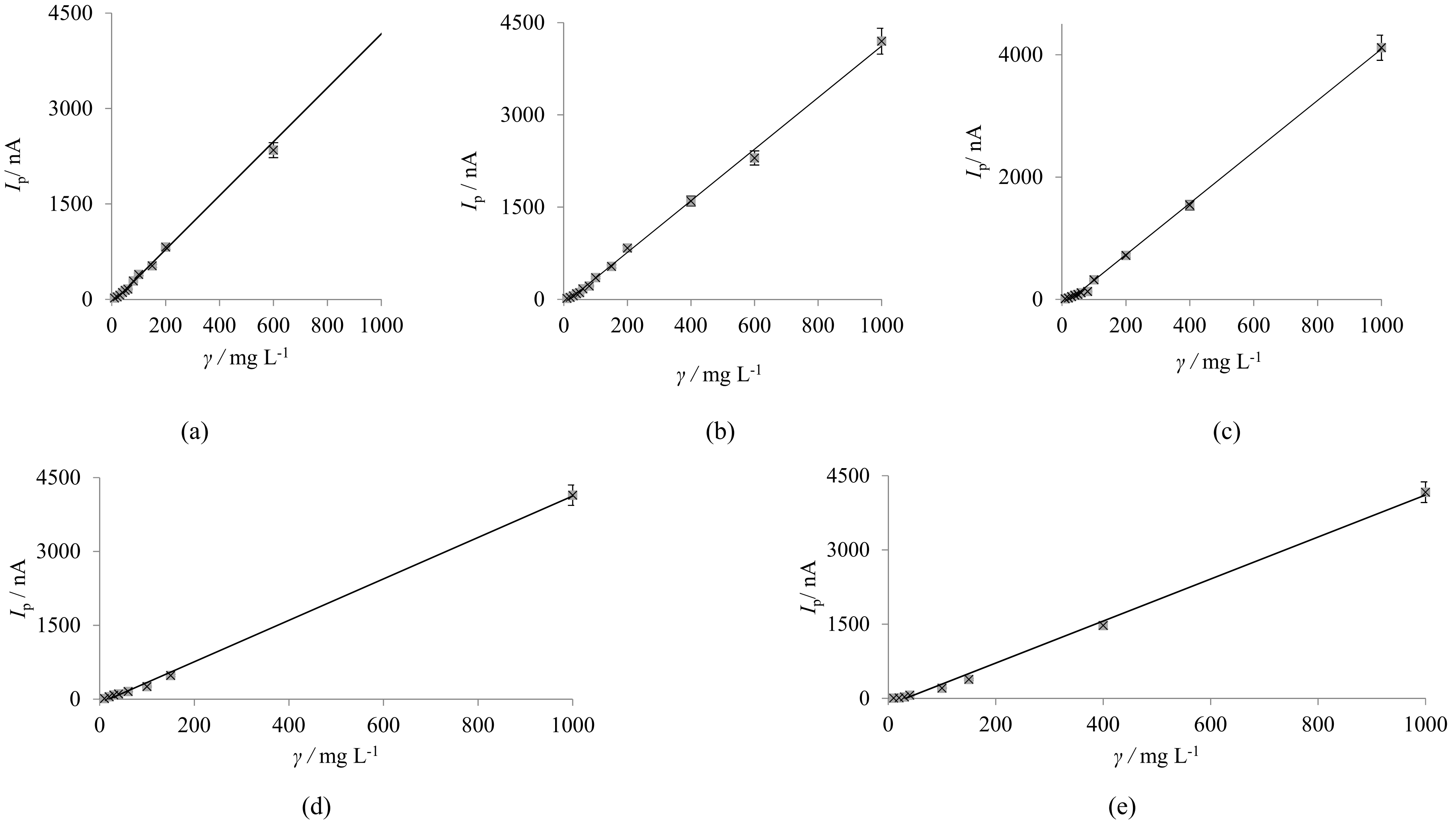

Figure S3. Variation of the oxidation peak current *I*p of the polyAgNPs@Sa with the γ mass concentration of polyAgNPs@Sa 5 to 1000 mg L^{-1} (a) 4, (b) 10, (c) 50, (d) 70 and (e) 100 mV s^{-1}. Other conditions are mentioned in the experimental section.

Table S1. Effect of the thickness of the formed film of poly-AgNPs@Sa. Other conditions are mentioned in the experimental section.

Number of Scans	Oxidation peak potential (V)	Oxidation peak current (μA)
1	0.324	1.260
3	0.349	1.650
5	0.274	2.690
7	0.249	1.950
10	0.274	0.254
15	-	-

Table S2. Effect of the mass concentration of AgNPs@Sa in their polymerization process on CPE. Other conditions are mentioned in the experimental section.

Mass concentration of AgNPs@Sa (mg L^{-1})	Oxidation peak potential (V)	Oxidation peak current (μA)
0.005	0.289	0.315
0.008	0.314	0.312
0.010^{-}	0.294	0.306
0.020	0.269	0.554
0.050	0.294	3.53
0.080	0.344	2.25
0.100	0.344	1.47
0.200	0.344	1.40
0.500	0.354	1.67
0.800	0.354	1.93
1.000	0.429	5.28
1.250	0.389	2.79
1.500	0.404	2.49

Table S3. Adsorptive transfer square wave stripping voltammetry frequency effect on the detection of mephedrone with the proposed electrochemical sensor. Other conditions are mentioned in the experimental section.

Frequency (Hz)	Oxidation peak potential (V)	Oxidation peak current (μA)
25	-	-
20	-	-
15	0.240	0.015
10	0.250	0.056
5	0.255	0.126

4	0.255	0.133
3	0.250	0.121
2	0.250	0.128
1	0.250	0,093

Table S4. Adsorptive transfer square wave stripping voltammetry pulse potential effect on the detection of mephedrone with the proposed electrochemical sensor. Other conditions are mentioned in the experimental section.

Pulse potential (mV s^{-1})	Oxidation peak potential (V)	Oxidation peak current (μA)
0.005	0.350	0.228
0.006	0.345	0.204
0.007	0.335	0.19
0.010	0.325	0.217
0.015	0.310	0.231
0.020	0.295	0.164
0.025	0.285	0.042
0.030	0.270	0.012
0.035	0.260	0.096
0.040	0.230	0.068
0.045	0.235	0.087
0.050	0.220	0.060
0.060	-	-
0.080	-	-
0.100	-	-
